# Preventing Premature Pre-Exposure Prophylaxis Discontinuation and Sexually Transmitted Infections Among Men Who Have Sex With Men (Project PEACH): Protocol for a Prospective Cohort Study

**DOI:** 10.2196/56096

**Published:** 2025-04-23

**Authors:** Amalia Aldredge, Derrius Carter, Candice A DeCree, Elliot V Gardner, Gina Bailey Herring, Oumaima Kaabi, Rebecca Moges-Banks, Rachel Valencia, Colleen Frances Kelley, Patrick Sean Sullivan

**Affiliations:** 1 Division of Infectious Diseases Department of Medicine Emory University Atlanta, GA United States; 2 Ponce de Leon Center Grady Healthcare System Atlanta, GA United States; 3 Department of Epidemiology Rollins School of Public Health Emory University Atlanta, GA United States

**Keywords:** PrEP, doxyPEP, HIV, sexually transmitted infections, men who have sex with men, pre-exposure prophylaxis, United States, prevention, syndemic, patient preferences, app-based, cohort study

## Abstract

**Background:**

There is an ongoing syndemic of HIV and sexually transmitted infections (STIs) in the United States, particularly among men who have sex with men (MSM). We have increasingly effective and diverse measures of prevention, including various types of pre-exposure prophylaxis (PrEP) for HIV prevention and doxycycline postexposure prophylaxis (doxyPEP) for STI prevention. As options expand, we need to understand how to use a combination of these strategies and other supports for MSM to best curb the syndemic.

**Objective:**

We designed a patient-preference trial to understand patient preferences for PrEP and doxyPEP, how preferences may change over time, and methods for preventing premature discontinuation of PrEP.

**Methods:**

We enrolled HIV-negative MSM in metropolitan Atlanta, Georgia. Participants could elect to take PrEP (daily or on-demand), doxyPEP, both, or neither, along with app-based support to evaluate for risk factors of discontinuation or behavioral changes that might affect their choice of prevention methods. Participants were able to switch prevention methods at any time. Oral PrEP and doxyPEP users are currently being offered quarterly in-person or at-home HIV, syphilis, gonorrhea, and chlamydia testing along with opportunities for motivational interviewing.

**Results:**

We enrolled individuals from November 2021 to September 2023. Among 240 participants, the median age was 30 (IQR 25-35), 63% (n=150) self-identified as non-Hispanic Black, and 69% (n=166) were insured. Most participants (n=144, 60%) elected to take daily PrEP plus doxyPEP, with a smaller proportion taking on-demand PrEP plus doxyPEP (n=34, 14%) or daily PrEP without doxyPEP (n=33, 14%).

**Conclusions:**

We designed an ongoing study to evaluate the preferences for PrEP and doxyPEP among MSM in metropolitan Atlanta. Enrollment was completed in 22 months and included a diverse cohort of MSM that will be followed longitudinally to evaluate prevention preferences over time. At baseline, most participants preferred to take a combination of daily PrEP and doxyPEP for HIV and STI prevention.

**Trial Registration:**

Clinicaltrials.gov NCT05072093; https://clinicaltrials.gov/study/NCT05072093

**International Registered Report Identifier (IRRID):**

DERR1-10.2196/56096

## Introduction

The US HIV epidemic continues to disproportionately impact men who have sex with men (MSM), who comprise 2% of the US population but account for about two-thirds of all new HIV diagnoses in the United States [1]. Epidemic modeling studies [2-4], our National HIV/AIDS Prevention Strategy [5], and the Centers for Disease Control and Prevention (CDC) Ending the HIV Epidemic initiative [6] all emphasize the critical role of pre-exposure prophylaxis (PrEP) in the national HIV prevention response. PrEP use is rising among men. Despite this, current estimates of coverage of PrEP among PrEP-eligible MSM fall short of the sustained 30%-50% coverage of PrEP among MSM that would be required to produce substantial decreases in new HIV infections [2,3]. PrEP use also varies widely geographically and by race. For example, in 2021, about 30% of PrEP-eligible men reported ever using PrEP in their lifetime [7]; in Ending the HIV Epidemic priority jurisdictions in metropolitan Atlanta, Georgia, less than 19% of PrEP-eligible MSM took PrEP [8].

Increasing rates of sexually transmitted infection (STI) diagnoses, independent of and concurrent with higher PrEP use [9], lead to concerns that men using PrEP may abandon condom use, contributing to greater increases in STIs among all MSM. Recent data from the CDC has shown a rise of 459% in the rate of cases of syphilis, 132% for chlamydia, and 96% for gonorrhea over the last 20 years [10], and although data from 2020 and 2021 may have been impacted by limitations of testing during the COVID-19 epidemic, there has overall been a steady increased rate of these STIs. The Southern United States has the highest rates of chlamydia and gonorrhea, and second highest rates of syphilis when compared to the West, Midwest, and Northeast, and rates of chlamydia and gonorrhea are highest in people who identify as Black or African American [10]. The recent introduction of postexposure prophylaxis with doxycycline (doxyPEP) to prevent STIs, which can decrease the risk of incident chlamydia, syphilis, and gonorrhea by up to 89%, 87%, and 55%, respectively [11], has been an encouraging prevention tool. The CDC recently released guidelines for the use of doxyPEP with a particular focus on offering it to MSM and TGW who have been diagnosed with an STI in the previous 12 months [12].

The syndemic of HIV and STIs is inherently intertwined given the shared mode of transmission, similar sociodemographic and structural risk factors, and as STIs can increase the risk of acquiring HIV [13]. Multiple prior studies have found that men on PrEP are more likely to discontinue taking PrEP after being diagnosed with an STI [14,15]; the exact reasons for this are unknown but thought to potentially be related to the change in sexual behaviors (eg, participants on PrEP noted they were engaged in more risky behaviors and thus had more STIs, so chose to discontinue it) and a marker of adherence to medical interventions. Given effective methods for the prevention of both HIV and STIs, they must be framed as a syndemic to provide comprehensive, patient-centered sexual health services to best support patients [16,17]. There are few data on PrEP uptake and persistence by PrEP modality and how preferences might change over time. In a sample of US MSM, on-demand PrEP was ranked as the most preferred mode of PrEP among nine possible routes of PrEP administration, and men reported statistically significantly higher intention to use on-demand oral PrEP than to use daily oral PrEP [18]. With the introduction of injectables and other forms of long-acting PrEP, more recent studies have shown that this may be an even more appealing option for individuals [19-21]. Many prior studies of PrEP have focused on recruiting individuals to start PrEP, but few have developed systems to mitigate PrEP discontinuations and prevent STI infections for MSM on PrEP.

The holistic focus relates importantly to implementation strategies because the barriers to persisting on PrEP are both structural (eg, distance to provider for follow-up visits, health insurance, and reimbursement mechanisms) and socio-relational (eg, attitudes of friends and family, relationship status, and perception of vulnerability to HIV) [22-25]. The barriers to starting PrEP (eg, lack of awareness of PrEP, barriers to care entry, stigma, and doubts about the efficacy or safety of PrEP) are not the same as the barriers to PrEP persistence (eg, lapse of insurance or assistance programs, difficulties with long-term adherence, changing sexual partners, frequency or networks, negative influence of friends or family, and concerns about new STIs) [26]. With the increasingly widespread availability of multiple modalities of PrEP, as well as doxyPEP as a novel method for STI prevention, it will be important to determine patient preferences in the combination of these prevention methods to best support services for individuals to prevent incident HIV and STIs.

We designed the Parrying the Pitfalls of PrEP: Preventing Premature PrEP Discontinuation and STIs Among MSM (Project PEACH) to offer a combination of sexual health prevention interventions. We recruited MSM without HIV living in metropolitan Atlanta, offered a bundle of sexual health prevention interventions, including multiple PrEP options and doxyPEP, and provided adherence and retention support through a mobile app and motivational interviewing. The purpose of Project PEACH is to understand which combination of sexual health interventions MSM will choose to use, how these might change over time, and to evaluate the determinants of PrEP persistence.

## Methods

### Design

Project PEACH is a prospective, observational cohort of 240 people assigned male sex at birth who have sex with other men (MSM) living in metropolitan Atlanta, Georgia. Recruitment of the cohort took place from November 2021 to September 2023. Participants are followed for 2 years with study assessments at baseline and months 4, 7, 12, 19, and 24. Study inclusion and exclusion criteria are shown in Textbox 1.

**Inclusion and exclusion criteria.**

**Inclusion criteria**

- Assigned male sex at birth
- Self-identification as a cisgender male
- Aged 18-45 years
- Lives in the metropolitan Atlanta area and not planning to move out of the area in the next 2 years
- Had at least one male anal sex partner in the 12 months before the baseline interview
- Able to complete survey instruments in English
- Provide 2 or more means of contact
- Own a cell phone with data service and willing to download and use a health-related study app

**Exclusion criteria**

- Positive HIV screening test at baseline visit
- Enrolled in another HIV prevention clinical trial

All participants were offered daily PrEP with tenofovir disoproxil fumarate (TDF) 300 mg/emtricitabine (FTC) 200 mg unless the participant had underlying chronic kidney disease with a creatinine clearance <50 mL/min or other contraindication for TDF/FTC per clinician review, in which case the participant was offered daily tenofovir alafenamide 25 mg/FTC 200 mg. All participants were also offered STI PEP with doxycycline 200 mg to be taken no longer than 72 hours after condomless sex. Participants who elected to use STI PEP were dispensed enough doxycycline to allow up to three weekly doses, but refills were readily available to participants if requested. If a participant was not interested in daily PrEP or planned to discontinue daily PrEP, they were offered on-demand PrEP. On-demand PrEP was offered with TDF/FTC and participants were directed to take two pills 2-24 hours before sex and to take an additional pill 24 hours after the first pill and a final dose 24 hours after that (“2-1-1”). TDF/FTC and tenofovir alafenamide/FTC prescriptions were provided for use at local pharmacies and navigation for insurance approvals and patient assistance plans for uninsured participants or participants with copays were provided. We amended our study protocol in May 2022 when injectable PrEP with cabotegravir became locally available. Patients were offered this option through local partner community organizations by providing them with information about clinics and instructing them to make an appointment, although injectable PrEP remained difficult to access throughout the study period. Participants were able to elect to start PrEP or STI PEP or change PrEP modality at each survey visit (months 4, 7, 12, and 19) or at any time through the study app.

All participants were provided with the study’s mobile SMaRT (Study Management and Retention Toolkit) phone app to support early identification of risks for PrEP discontinuation and offer alternative prevention methods, to identify any side effects from medications, to provide information about STI PEP and document use patterns of on-demand PrEP and STI PEP, and to support easy linkage to support services for PrEP counseling and addressing concerns or questions about STI PEP. Participants are able to complete monthly (daily PrEP and injectable PrEP users) and weekly (on-demand PrEP, STI PEP, and first 2 weeks for daily PrEP users) short surveys within the SMaRT app, which also provides links to web-based study assessments at months 4, 7, 12, 19, and 24.

### Recruitment, Enrollment, and Retention

Participant recruitment was conducted through multiple sources. We used a modified venue-day-time screening approach using data from past studies to prioritize venues with historically high numbers of MSM who were HIV-negative [27,28]. We offered a US $5 incentive to take the study screener at in-person recruitment events. We used dating apps and social media with targeted advertisements to adult MSM in Atlanta and offered US $20 incentives to current study participants who shared our institutional review board (IRB)–approved study ads on their social media. Our initial recruitment plan was to exclude participants already on PrEP, but given limitations in enrollment during the COVID-19 pandemic, we expanded eligibility in May 2022 to include participants currently prescribed PrEP. NIH recruitment goals were set to include ≥70% of participants who identify as a racial or ethnic minority, including ≥10% of all participants identifying as Hispanic.

Participants who were eligible for the study based on the initial screening questionnaire were scheduled for an enrollment (baseline) visit at the community-based Programs, Research, & Innovation in Sexual Minority Health (PRISM) Health Research Clinic. This clinical site is located 1.5 blocks from public transportation, and ride-share services were provided to participants with limited transportation opportunities or long commutes. The enrollment visit consisted of an informed consent process, a computer-assisted self-interview behavioral survey, HIV and STI testing, training on using the study app, and a counseling session to include pre- and posttest HIV prevention information and a discussion about PrEP and STI PEP options. Participants who completed an enrollment visit were compensated US $125 regardless of whether they subsequently participated in the study. Participants with a positive HIV screening test at baseline or any point during the study were linked to HIV care.

To maximize retention, we used several strategies. We hired a study staff member dedicated exclusively to retention who collaborated with the study team to promote retention among participants. The SMaRT mobile app was customized for this study to allow participants to schedule, reschedule, receive reminders of, and cancel study appointments; communicate securely with study staff; and update contact information from within the Health Insurance Portability and Accountability Act (HIPAA)-compliant app. Participants were reminded about their follow-up visits through the app messaging system or their preferred means of contact both 3 days and 1 day before the scheduled follow-up date. We also sent reminders for surveys up to one week prior and up to 20 days after the target date through the SMaRT app. If we lost contact with participants (missed surveys or appointments and were nonresponsive to contact requests), we obtained consent to use for-fee public-use databases (eg, Lexis-Nexis) to locate participants; this has been used by us in previous studies to relocate participants who were lost to follow-up [29,30]. For participants who could not be contacted, after IRB approval, staff first attempted to contact emergency contacts, and if unsuccessful, attempted to link the identities of participants to registries of known decedents using a statewide data set (vital records) and a nationwide data set (National Death Index).

### Study Procedures

All participants who elected to use oral PrEP or STI PEP received brief weekly surveys for the first two weeks after starting medications, with a US $5 incentive provided for each survey. After this period, daily PrEP and injectable PrEP users received monthly short surveys to assess risk for discontinuation (US $10 incentive for each survey) whereas participants who opted for on-demand PrEP or STI PEP continued to receive weekly short surveys (US $5 incentive for each survey). All participants were sent computer-assisted self-interview surveys at months 4, 7, 12, 19, and 24, and encouraged to attend in-person follow-up visits at 12 and 24 months (US $40 at 4, 7, and 19 months; US $100 at 12/24-month survey and study visit) and additional telehealth or in-clinic visits if the risk for PrEP discontinuation was identified by surveys. Daily or on-demand PrEP users or STI PEP users had the option for in-person or home-testing for HIV/STIs every 3 months for clinical monitoring of their medication use. For injectable PrEP users, all clinical monitoring was done outside the study by a local provider of the participants’ choosing.

### Motivational Interviewing

Risk factors for discontinuing PrEP were developed using data from a previous cohort of MSM in Atlanta (Textbox 2) [14,31,32]. When a participant reported any of these risk factors for discontinuing PrEP on a monthly survey, they were invited to schedule a discussion with a peer navigator with the intention of identifying possible risks for PrEP discontinuation, minimizing discontinuation of PrEP, or offering another prevention option. Triage sessions were client-centered, using an approach grounded in motivational interviewing. By focusing on the last time that the client was confident about being on PrEP (eg, think back to when you were taking your medication every day, what was going on in your life? Where were you living? Who were you dating?), the peer navigators were able to better understand the holistic context of the current risk factor. Based on this comprehensive understanding of needs and the monthly screening data, trained peer navigators used motivational interviewing tools to help participants problem-solve based on the needs identified and worked with them to develop a plan to address those concerns while maintaining PrEP, if appropriate. Clinical concerns (eg, side effects) were referred to a PrEP clinician for discussion.

**Risk factors for discontinuing pre-exposure prophylaxis (PrEP).**

- Issues with remembering to take PrEP
- Stigma (ie, judgment from friends or family)
- The side effects made me feel bad
- Did not want to take a pill every day
- Did not think I was at risk anymore (decrease in risky behavior)
- Having sex less frequently
- Change in sex partner
- Time conflict
- Increase in risk behavior
- Housing issues
- Cannabis use
- Depression
- Anxiety
- Had bad experiences (eg, clinic not open at convenient times)
- Too much going on in my life right now

### Laboratory Specimens

Laboratory testing was done at the research clinic or remotely by the participant at home using self-collection specimen methods and differed by prevention method choice as described in Table 1. Clinical monitoring of injectable PrEP use was done by the local provider who administered the medication. Any positive HIV/STI test results were provided to participants over the phone by a study staff member who could refer participants to study-paid treatment for STI infections at a local community-based health clinic, or to participant-paid treatment through a provider of their choosing. Negative test results were delivered through the HIPPA-compliant study app. To minimize bias in self-report data on substance use, we collected urine drug screening in our on-site clinical laboratory improvement amendments-waived laboratory at baseline, 12, and 24 months using the Ten Panel Integrated EZ Split Key Cup Drug Test. We attempted to examine Neisseria gonorrhoeae resistance patterns among men who acquired the infection during follow-up by having them return for Neisseria gonorrhoeae culture prior to treatment with compensation of US $100; however, we discontinued this when none of the isolates grew in culture.

**Table 1:** Study design of testing and surveys by group.

Test or survey type	Group of participants		
	If elect daily oral PrEP^a^	If elect daily oral PrEP or injectable PrEP	If elect on-demand PrEP or doxyPEP^b^
HIV Ag/Ab, RPR^c^, urine/throat/rectal GC^d^/CT^e^ PCR^f,g,h,i^	Baseline and every 12 months	Baseline and every 12 months	Baseline and every 3 months
Web-based computer-assisted self-interview surveys	Months 4, 7, 12, 19, and 24	Months 4, 7, 12, 19, and 24	Months 4, 7, 12, 19, and 24
Serum creatinine	Baseline and every 12 months	N/A^j^	N/A
Hepatitis B surface antigen	Baseline	N/A	N/A
Survey of PrEP discontinuation risk	N/A	Monthly	N/A
Survey of PrEP use and discontinuation risk	N/A	N/A	Weekly

^a^PrEP: pre-exposure prophylaxis.

^b^doxyPEP: doxycycline postexposure prophylaxis.

^c^RPR: rapid plasma reagin.

^d^GC: *Neisseria gonorrheae*.

^e^CT: *Chlamydia trachomatis*.

^f^PCR: polymerase chain reaction.

^g^HIV Ag/Ab screening done in-person via INSTI (a clinical laboratory improvement amendment-waived HIV rapid test); if positive or indeterminate or if someone was initiating PrEP/ sexually transmitted infection PrEP, we sent a blood sample to Quest for commercial fourth-generation HIV testing. If HIV Ag/Ab screening was done remotely, patients collected a dried blood spot card and were sent to a laboratory for fourth-generation HIV testing specifically for dried blood spot protocol.

^h^Participants self-collected pharyngeal, urine, and rectal samples for *N gonorrhea* (GC) and *C trachomatis* (CT) using Abbott Real Time GC/CT assay.

^i^Serum samples for a US Food and Drug Administration–approved syphilis RPR test were either collected in-person at the clinic by a phlebotomist or remotely via participant fingerstick blood sample. If RPR is reactive on home tests, participants were asked to come to the clinic to have an RPR titer drawn.

^j^N/A: Not applicable.

### Planned Analyses

In Table 2, we have outlined the proposed analyses for this study. We will evaluate the rate of PrEP uptake, rate of doxyPEP uptake, rate of PrEP discontinuation, rate of doxyPEP discontinuation, and rate of incident STI and HIV diagnoses by prevention choice. We will describe use patterns of PrEP modality and STI PEP over the study period including changes in use. We will also describe the proportion of participants who initially chose each method of PrEP and the proportion who switched method of PrEP during the study period. We will describe the utilization of various support systems offered. Finally, we will compare incident STI diagnoses (gonorrhea, chlamydia, syphilis) at 12 and 24 months in this cohort to a historical control group to evaluate the effect of STI PEP.

**Table 2:** Proposed outcome measures and planned analyses.

Outcome measure	Planned analyses
PrEP^a^ uptake	Descriptive analysis—prevalence
DoxyPEP^b^ uptake	Descriptive analysis—prevalence
PrEP discontinuation	Proportion of PrEP users discontinuing, by group Time to discontinuation by Cox Proportional Hazards model (if sufficient events for analysis)
DoxyPEP discontinuation	Proportion of doxyPEP users discontinuing, by group Time to discontinuation by Cox Proportional Hazards model (if sufficient events for analysis)
Change in STI^c^ prevention modality	Descriptive analysis with alluvial plots or heat maps
Utilization of support systems (eg, motivational interviewing and application)	Descriptive analysis—prevalence
Time to incident HIV diagnoses	Cox proportional hazards model
Time to incident chlamydia diagnoses^d^	Cox proportional hazards model
Time to incident gonorrhea diagnoses^d^	Cox proportional hazards model
Time to incident syphilis diagnoses^d^	Cox proportional hazards model

^a^PrEP: pre-exposure prophylaxis.

^b^DoxyPEP: doxycycline postexposure prophylaxis.

^c^STI: sexually transmitted infection.

^d^Plan to compare incident STI diagnoses at 12 and 24 months to historical Atlanta data over the same time period using a denominator of men who have sex with men.

### Ethical Considerations

The study was approved by the Emory IRB (STUDY00000608). Informed consent was provided by participants at the time of enrollment and participants were given the option to opt out of the study at any time. The data obtained were all anonymized using a unique patient identifier number. Participants were compensated for their time as outlined above.

## Results

**Figure 1:**
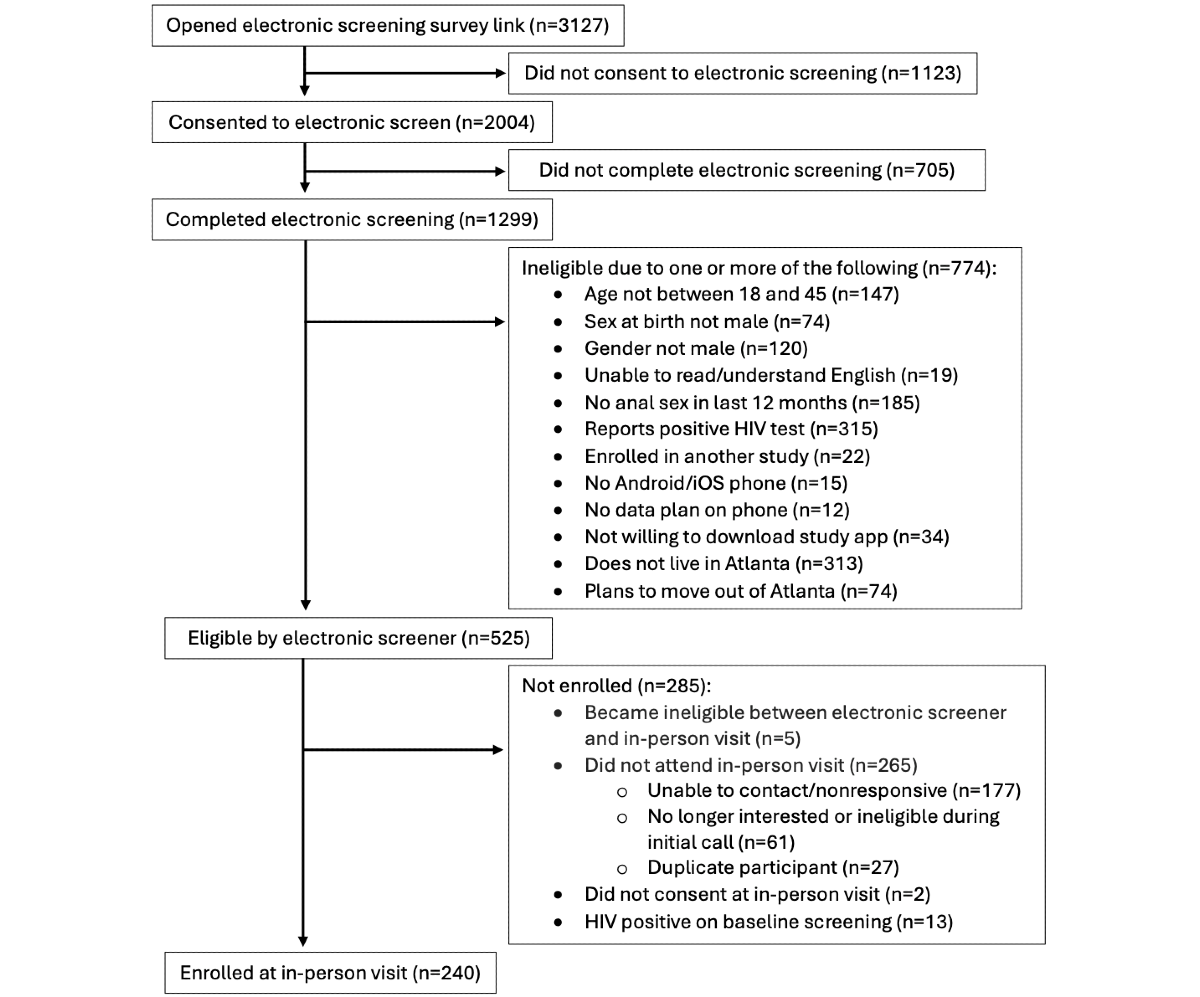
Flowchart of participants included in the analysis and reasons for exclusion at each step in the enrollment process. This was an adaptive design, patient preference trial in which men who have sex with men in Atlanta, Georgia from 2021 to 2023 were able to choose a combination of HIV PrEP and STI postexposure prophylaxis (doxyPEP) that was best for their needs. Individuals who completed electronic screening but were ineligible for the study could have been excluded for multiple reasons. doxyPEP: doxycycline postexposure prophylaxis; PrEP: pre-exposure prophylaxis; STI: sexually transmitted infection.

The study was approved by the Emory University IRB in May 2021 and launched in September 2021. Of the 1299 people who completed electronic screening, 525 people were eligible for the study (Figure 1). We enrolled 240 men in Atlanta, Georgia, over the course of 21 months from November 2021 through September 2023. Median age was 30 (IQR 25-35), 63% (n=150) self-identified as non-Hispanic Black, 12% (n=29) as Hispanic, 19% (n=46) as White, and 6% (n=15) as Other. A total of 31% (n=74) were uninsured at baseline (Table 3).

Laboratory measurements at baseline are shown in Table 4. Treponemal antibody results are presented below, which could represent past or current infection. For primary analysis, cases of syphilis will be individually adjudicated.

**Figure 2:**
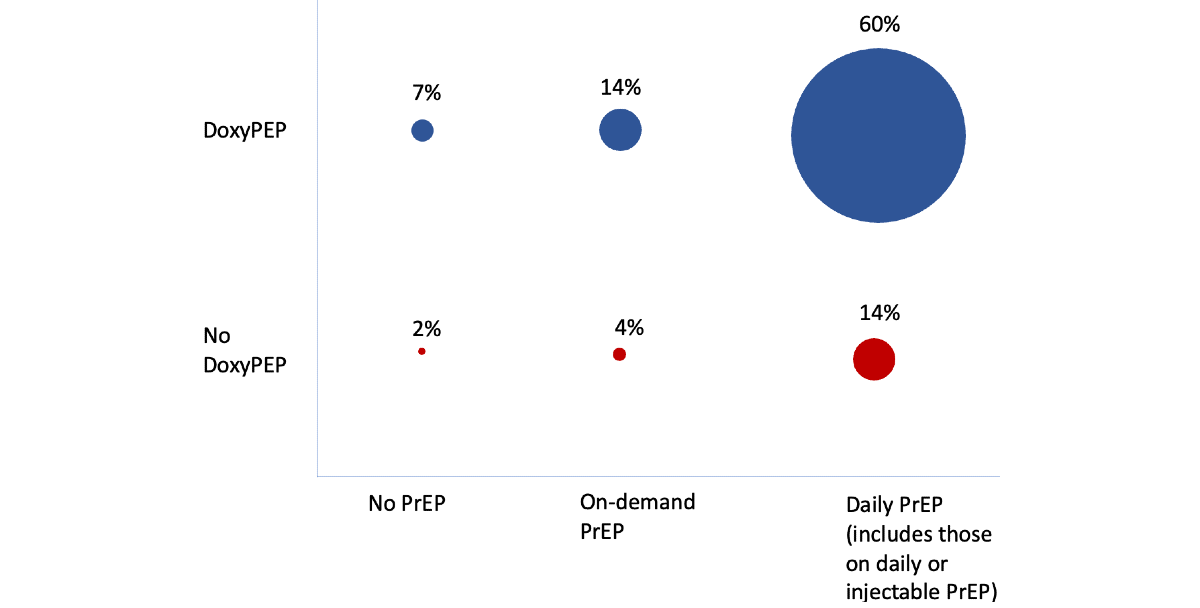
Choices at enrollment of type of PrEP and doxyPEP. A majority of participants (n=144, 60%) preferred to use both daily PrEP and doxyPEP together. The next most common choices were either daily PrEP alone or on-demand PrEP with doxyPEP. The above proportions are to scale and are across all categories. Proportions do not add up to 100% due to rounding. doxyPEP: doxycycline postexposure prophylaxis; PrEP: pre-exposure prophylaxis.

At first visit, 57% (n=136) elected for daily PrEP and STI PEP, 14% (n=34) on-demand PrEP and STI PEP, 13% (n=31) daily PrEP only, 7% (n=17) STI PEP only, 4% (n=9) on-demand PrEP only, 3% (n=8) injectable PrEP and STI PEP, 2% (n=5) app only, and 1% (n=2) injectable PrEP only (Figure 2). Of note, although injectable PrEP was offered through the study starting in May 2022, the first date that a study participant received injectable PrEP was March 2023.

**Table 3:** Baseline demographics of participants.

Participant characteristics		Total participants (n=240)	PrEP^a^ + DoxyPEP^b^ (n=177)	PrEP only (n=42)	DoxyPEP only (n=16)	App only (n=5)
Age (years), median (IQR)		30 (25-35)	31 (25-35)	29 (24-33)	32 (28-37)	24 (23-28)
Race/ethnicity, n (%)						
	Black, non-Hispanic	150 (63)	108 (61)	28 (67)	12 (75)	2 (40)
	Hispanic	29 (12)	22 (13)	4 (9)	1 (6)	2 (40)
	White, non-Hispanic	46 (19)	34 (19)	8 (19)	3 (19)	1 (20)
	Other	15 (6)	13 (7)	2 (5)	0 (0)	0 (0)
Insured, n (%)		166 (69)	120 (68)	33 (79)	9 (56)	4 (80)

^a^PrEP: pre-exposure prophylaxis.

^b^DoxyPEP: doxycycline postexposure prophylaxis.

**Table 4:** Baseline laboratory data of participants.

Baseline participant laboratory measurement			Total participants (N=240), n (%)
Hepatitis B surface antigen-positive			0 (0)
Creatinine clearance <60 mL/min			2 (0.4)
**Chlamydia PCR^a^ test positive**			
	Pharyngeal	5 (2)	
	Rectal	12 (5)	
	Urine	8 (3)	
**Gonorrhea PCR test positive**			
	Pharyngeal	8 (3)	
	Rectal	12 (5)	
	Urine	1 (0.4)	
**Syphilis test results**			
	Treponemal antibody positive^b^	41 (17)	

^a^PCR: polymerase chain reaction.

^b^Indicates past or current infection.

## Discussion

### Principal Findings

Given the ongoing HIV and STI syndemic, there is a great need to leverage available tools to help prevent these infections. We designed this study to investigate the preference for HIV and STI prevention strategies and support mechanisms among MSM in Atlanta, Georgia. After the enrollment of 240 men, we found that their initial choice of prevention was typically daily PrEP combined with doxyPEP. Of all prevention methods, combining PrEP with doxyPEP was more desirable than doxyPEP alone. To our knowledge, this is the first study to evaluate the preference for a combination of PrEP and doxyPEP. Participants will be followed longitudinally for 24 months to evaluate the persistence of these prevention interventions and changes in preferences over time. Strengths of this study include that we have recruited a racially and ethnically diverse cohort of participants with two-year longitudinal follow-up, and its novel design to evaluate how patient preferences change over time.

In several prior studies of preference for PrEP modality, individuals have prioritized PrEP effectiveness and low-cost options; as more long-acting PrEP options become available, recent studies have demonstrated that this is often desirable, but that preference for long-acting PrEP may differ between groups [21,33,34]. During enrollment in this study, long-acting injectable PrEP with cabotegravir was approved in the United States for HIV prevention. Although we incorporated this change into our study protocol, there were delays in clinics being able to offer this option to patients, leading to a few participants selecting this as their initial choice. Given this, although it appeared very few people chose injectable PrEP initially, this is likely not representative of true choices given the initial lack of availability. We anticipate that individuals may change their preferences for using PrEP and STI prevention methods based on factors like sexual practices and that motivational interviewing through this study can assist participants persist or reengage in using prevention methods.

### Limitations

There are a few limitations to this study. Although initial results suggest a preference for daily oral PrEP combined with doxyPEP, injectable PrEP was not yet available at the time of enrollment for most individuals; this preference may be different in the era of injectable PrEP. Individuals were eligible for the study only if they lived in metropolitan Atlanta, so conclusions from this study may be different for populations in different geographic areas. Although the study was open to all individuals regardless of insurance status, nearly 70% of participants were insured, which may have affected the choice of STI prevention method. Given that this is an observational study, there is potential for unmeasured bias.

### Conclusions

We plan to evaluate the preferences of combination PrEP and doxyPEP over the course of 2 years and describe trends including changes in use over time. We also plan to evaluate the impact of support tools like motivational imaging on the persistence of sexual health prevention methods. Bundling PrEP and doxyPEP in a way to be most desirable and acceptable to MSM, along with determining what support tools augment persistence, will be imperative to curb the HIV and STI syndemic, which is particularly pronounced in the southern United States. We plan to present the findings of our work at community advisory board meetings, community-oriented presentations, and through peer-reviewed publications, with the goal of ensuring patients are supported in choosing a combination of sexual health prevention methods that are best for their needs.

## Abbreviations

CDC: Centers for Disease Control and Prevention
doxyPEP: doxycycline postexposure prophylaxis
FTC: emtricitabine
IRB: institutional review board
MSM: men who have sex with men
PrEP: pre-exposure prophylaxis
SMaRT: Study Management and Retention Toolkit
STI: sexually transmitted infection
TDF: tenofovir disoproxil fumarate

## Data Availability

The data sets generated during or analyzed during this study will be available after publication of the primary results from the corresponding author on reasonable request with an executed data use agreement.
